# "If you don't believe it, it won't help you": use of bush medicine in treating cancer among Aboriginal people in Western Australia

**DOI:** 10.1186/1746-4269-6-18

**Published:** 2010-06-23

**Authors:** Shaouli Shahid, Ryan Bleam, Dawn Bessarab, Sandra C Thompson

**Affiliations:** 1 Centre for International Health, Curtin University, GPO Box U1987, Perth WA 6845, Australia; 2 Curtin Health Innovation Research Institute, Curtin University, GPO Box U1987, Perth WA 6845, Australia; 3 Combined Universities Centre for Rural Health, University of Western Australia, PO Box 109, Geraldton WA 6531, Australia

## Abstract

**Background:**

Little is known about the use of bush medicine and traditional healing among Aboriginal Australians for their treatment of cancer and the meanings attached to it. A qualitative study that explored Aboriginal Australians' perspectives and experiences of cancer and cancer services in Western Australia provided an opportunity to analyse the contemporary meanings attached and use of bush medicine by Aboriginal people with cancer in Western Australia

**Methods:**

Data collection occurred in Perth, both rural and remote areas and included individual in-depth interviews, observations and field notes. Of the thirty-seven interviews with Aboriginal cancer patients, family members of people who died from cancer and some Aboriginal health care providers, 11 participants whose responses included substantial mention on the issue of bush medicine and traditional healing were selected for the analysis for this paper.

**Results:**

The study findings have shown that as part of their healing some Aboriginal Australians use traditional medicine for treating their cancer. Such healing processes and medicines were preferred by some because it helped reconnect them with their heritage, land, culture and the spirits of their ancestors, bringing peace of mind during their illness. Spiritual beliefs and holistic health approaches and practices play an important role in the treatment choices for some patients.

**Conclusions:**

Service providers need to acknowledge and understand the existence of Aboriginal knowledge (epistemology) and accept that traditional healing can be an important addition to an Aboriginal person's healing complementing Western medical treatment regimes. Allowing and supporting traditional approaches to treatment reflects a commitment by modern medical services to adopting an Aboriginal-friendly approach that is not only culturally appropriate but assists with the cultural security of the service.

## Introduction

*Indigenous Peoples' concept of health and survival is both the collective and individual inter-generational continuum encompassing a holistic perspective incorporating four distinct shared dimensions of life, which are the spiritual, intellectual, physical, and emotional. Linking these four fundamental dimensions, health and survival manifests itself on multiple levels where the past, present and future co-exist simultaneously*[1]

The holistic health care system has been practiced by people from different ethnic and cultural background worldwide[2]. While these health systems in various parts of the world share certain characteristics that distinguish them from biomedicine, approaches to health and healing are diverse and changing over time. Nevertheless, some commonalities can be distinguished [3]. In Australia (as elsewhere), Aboriginal people have relied on plants for many of their needs, including as a medicine in treating their ailments[4]. Aboriginal and Torres Strait Islander people are the Indigenous people and original inhabitants of Australia. In this paper, 'Aboriginal' has been used to refer to the Aboriginal and Torres Strait Islander people who are traditional inhabitants and Indigenous people of Australia. 'Indigenous' has been used when we refer to descendents of the native or first nation inhabitants of other countries prior to European colonisation. Since colonisation, the lifestyles of Aboriginal Australians have endured significant change through dispossession of land, social disruption, racism, cultural suppression and discriminatory government policies[5]. Consequently, people's usage of plants and maintenance of traditional cultural beliefs and practices, including traditional medicine and healing practices[6] have varied according to the impact of colonisation on their connection to country and culture. Previous research confirming the use of traditional medicines[6,7] by Indigenous people have recognized that failure to understand and communicate about such usage may result in patients' dissatisfaction and non-compliance with existing bio-medical treatment services[8,9]. This however, has not translated to mainstream health service providers who appear to have little recognition and acknowledgement of the belief in and use of traditional healing practices and medicines by Indigenous patients[10,11].

This analysis arose in the context of a research project that aimed to explore the beliefs, understanding and meaning of cancer to Aboriginal people in Western Australia (WA) and their experiences with cancer services. Although the use of bush medicine was not a particular focus of the study a number of participants raised the issue of using traditional healers and bush medicine for cancer during their interviews.

This paper provides an overview of the use of bush medicine and traditional healing amongst Aboriginal Australians for their treatment of cancer and the meaning attached to it and argues for health service providers to recognize its importance in the life of Aboriginal people, especially during consultation. Effective and culturally sensitive health care provision for Indigenous communities requires respect for patients' beliefs and practices of healing. Healing is 'a process that brings parts of one's self (physical, emotional, mental and spiritual) together' which 'can result in an integrated and balanced whole self'[12]. Thus, it includes ceremonies, traditions, values and ideas related to Indigenous culture[12]. There exists limited written information on such healing traditions in Aboriginal Australian communities as secrecy and mysticism are attached to the use and origin of such practices and medicines.

## Aboriginal Australians and Cancer

In recent years there has been an increased priority given to Aboriginal cancer in mainstream health[13]. When compared to the difference in life expectancy between Aboriginal and non-Aboriginal Australians[5], cancer is now one of the leading causes of death amongst the Aboriginal population[14,15]. For many years cancer was not prioritized as a health issue, primarily because of the low life-expectancy of Aboriginal Australians and more immediate and obvious health problems. Overall, the literature suggests that the incidence of various cancers is lower in surveillance research data[16,17] in part due to the misclassification of Aboriginal status in cancer registries[18]. Additionally, as a small minority of the total Australian population[16], it is difficult to provide statistically significant figures for Aboriginal Australians. Selected data are available for some cancers, including a five times higher mortality rate for women's cervical cancer[16], and almost two times higher incidence rate of liver cancer than that of non-Aboriginal Australians. The limited available data highlights a need for more attention to be given to Aboriginal cancer.

Aboriginal Australians with cancer are twice as likely to die from the disease than non-Aboriginal Australians[19]. This could be due to the fact that Aboriginal people are diagnosed later than their non-Aboriginal counterparts; have poorer continuity of care and a lower compliance with treatment[15,20]. They also suffer from cancers which generally have a poor prognosis but are largely preventable[15]. The late diagnosis has been attributed to a general fear of check-ups and screenings[21,22]. Some of the traditional beliefs surrounding sources of illness attribute the cause of disease to acts of spiritual punishment, sorcery, payback, taking something from country or trespassing on a significant site[23,24]. Payback and sorcery may be bestowed upon those who do not fulfil social obligations or break a moral taboo[25]. These forms of cultural punishments could explain the reluctance of some Aboriginal people to seek early intervention for their illness due to a fear of community shame. Although such beliefs are primarily held in remote and traditional areas, these views are also held by urban and metropolitan Aboriginal populations[25,26].

Late diagnosis and discontinuity of treatment can also occur due to the fact that the hospital setting is a source of social unease for Aboriginal patients[25,27]. The practitioner's waiting room can present as a foreign environment where Aboriginal patients may experience themselves as outsiders in a sterilized, Western clinical setting. Additionally the thought of a 'private consultation' singles out the patient and creates further discomfort and shame. As many studies [6,25,27,28] have pointed out, shame (a violation of cultural or social values so it is possible to feel ashamed of thought or behavior that no one knows about) is a unique and powerful emotion to Aboriginal Australians. Another form of shame can come from gender-specific issues and the resistance to being examined or having to talk about symptoms with someone of the opposite sex[25,27]. Shame is also associated with cancer because many Indigenous people feel it is a 'white man's disease' [23,28-30]. This sentiment may discourage Indigenous people from believing they are at-risk of cancers[23] or may prevent them disclosing their illness to others[24,28].

In Indigenous belief systems, health is closely related to what tasks one can perform in society [25,31], hence, when treatment takes the patient away from performing, it is seen as a step backward in health. Treatments that make the patient sick, such as chemotherapy, are therefore deemed to be undesirable[31]. Furthermore, the holistic health framework associated with Indigenous health belief purports that spiritual, physical, and emotional factors are essential to ones' interconnected wellbeing[6,30]. This alternate view is in conflict with the biomedical position which focuses on physical health[25], thus creating the opportunity for miscommunication and misunderstanding between Indigenous patients and Western health service providers that cannot be overstated[27,32] to occur. Miscommunication can also occur due to the ethnocentric setting of the clinic, whereby the providers of care have different cultural nuances - beliefs, mannerisms, language and body language- to their patients, leading to misinterpretation by either patient or clinician.

Many Indigenous languages do not have a word for cancer, making it difficult to conceptualize[23]. This emphasizes the belief that it is a 'white man's disease' that only came about after colonization. Whether this is true or whether it was never labelled as cancer until colonization is unknown; however, many signs point to the fact that the change in Indigenous lifestyle from traditional to Westernized has increased the risk of cancer[33].

## Methods

This project was developed in response to a need identified by health service providers for greater understanding of Aboriginal Australians' beliefs, understanding and experiences of cancer, cancer care and treatment[34]. Aboriginal people have also argued for their health needs to be better understood by the western health system. The research was approved by the Western Australian Aboriginal Health Information and Ethics Committee (WAAHIEC), the Human Research Ethics Committee of Curtin University and The Royal Perth Hospital and Sir Charles Gairdner Hospital Ethics Committees. An Aboriginal Reference Group (ARG) was established, involved and consulted throughout the study period.

Inclusion criteria for the study involved Aboriginal adults who were cancer patients, survivors, family members of people with cancer or people who died from cancer who are or were intimately involved in another's cancer journey. Detailed description of the processes and methods for the study are published elsewhere[35]. In short, recruitment occurred through the networks of the researchers and reference group members, via health professionals in primary or tertiary care, through relevant Aboriginal Health Services or other local support agencies. Thirty-seven [Demographic details are presented in Table 1] in-depth open-ended interviews were conducted with Aboriginal participants (including some Aboriginal health service providers) in Perth (urban) and in one rural and two remote areas of WA between March 2006 and September 2007. Participants included patients and family members who were diagnosed or died from different types of cancer. Most of them were diagnosed with breast cancer (N = 11) followed by lung (3), cervical (4), bowel (2), throat (2), head and neck (2), pancreas (1), leukaemia (1), ovarian (1), mesothelioma (2) and melanoma (1). All participants spoke English and gave written informed consent. However, English was not the first language for a few participants, especially those who were recruited from remote communities. Participants were asked about their experience (either in their own life or that of their family) with cancer. Interviews were audio-recorded and transcribed verbatim. Thematic analysis was undertaken where transcripts were reviewed by two researchers independently and the material read repeatedly to derive the key themes. Thematic analysis is independent of epistemology and theory providing a flexible and useful research tool which can give a detailed as well as complex description of the data[36].

**Table 1:** Characteristics of Study Participants

Aboriginal Participants (n = 37)		Aboriginal participants who made mention about bush medicine (n = 11)	
**Area of Residence**		**Area of Residence**	
Urban participants	15	Urban	6
Rural participants	9	Rural	3
Remote participants	8	Remote	2
**Category of Respondent**		**Category of Participants**	
Patients	14	Patients	4
Family Members	16	Family Members	4
Health Service Providers	07	Health Service Providers	3
**Sex**		**Sex**	
Male	8	Male	2
Female	29	Female	9
**Age (years)**		**Types of cancer treated**	
30-39	5	Breast	3
40-49	19	Cervical	2
50-59	9	Head and Neck	1
60+	4	Lung	2
		Unknown	1
		**General mention about bush medicine**	2

## Findings

Of the thirty-seven interviews conducted, twenty-two mentioned bush medicine in some form. Many were minor references that did not encapsulate clearly the connection between bush medicine and cancer treatment. The eleven interviews that made significant mention of bush medicine (seven were prompted and four were spontaneous) are the focus for this paper; thus, the comments and themes elaborated reflect only a proportion of Aboriginal people's perspectives on this issue. The themes have been organized to explore two questions: i) what were the key factors for which Aboriginal patients chose to use bush medicine and ii) what factors influenced their decision not to use it. Subthemes are described below:

### Reasons for using bush medicine for cancer

Respondents who mentioned using bush medicines saw it as a preventive means to cope with the stress of cancer and believed that the healing powers could help to cure and relieve the anxiety and conditions of cancer.

Relieves stress: *"... it gets rid of all your internal stress"*

The belief that stress can cause cancer was brought up by many of the respondents. The views of a number of participants were encapsulated in a comment by one participant who saw cancer arising as a flow on effect of the disruption and stress following colonisation:

"*One minute, Aboriginal people had land and [then the] 1905 Act... see all those land taken away. ...so, that causes a lot of stress... the stolen generation... stress. We know... people were in stress and depression... that sort of things can cause cancer." *[Urban male participant]

Related to this was the idea that bush medicine reduces the risk of cancer. Bush medicine was regarded as a preventive measure as it helped to release stress, making the person stronger from the inside:

"*What happens is... it's a bush... or root... that you boil it up... and... it's a browny... it's got like a barky taste like a woody taste... But there is something in it... that is good for insides, just as a cleanser. Makes all your body organs healthy and strong, it gets rid of all your internal stress." *[Urban male participant]

Another participant talked about maintaining her longstanding belief in bush medicine and using it even after being diagnosed with cancer. She explained that a great deal of Aboriginal people were naive about cancer and got stressed when they heard the diagnosis. From others' stories it was clear that, to them, bush medicine could help in releasing the burden of their illness.

The 2002 National Aboriginal and Torres Strait Islanders Social Survey showed that Aboriginal people over the age of 18 were 1.5 times more likely to have reported experiencing a life stressor. Lurking in the collective memory of Aboriginal Australians is the legacy of child removal (the "stolen generation") and other historical mistreatments experienced in their recent past with its devastating effect on cultural practices and living conditions, including creating barriers to the development of social capital within Aboriginal communities[37].

A connection to spirituality and holistic health worldview: *"Healing is mental, emotional and spiritual as well"*

For some of the participants the application of bush medicine was not only seen as relieving stress but was also seen as an enabler in maintaining their connections and beliefs on culture, ancestors and spirituality. The practice of bush medicines confirmed and supported participant's cultural beliefs and attitudes that conformed to Aboriginal understandings and epistemologies of health and wellbeing as holistic. Engaging with bush medicines and the associated healing rituals that accompany its use is spiritually significant to Aboriginal people whose identity and connection is embedded in their relationship to the land. The relationship that Aboriginal people have with the land is sacred and related to their concept of health, wellbeing and healing[27]. Two excerpts clearly illustrate this connection:

"*Yeah, their spirituality is always there; they link bush medicine with the land, but it is very hard to get, because there's not many people who go out and get it. You get it from certain trees and what-have-you. But that belief that trying bush medicine will heal them is still there." *[Urban female participant]

Consistent with several other interviewees, this participant would not go into particular detail about where you can get bush medicine and what it is. This keeps the spiritual mysticism alive.

"*An old lady came up there with a bottle. I said, I can't eat for six months... can't swallow anything. She said, "You drink it, and you would get better." And I believed that. And it's gone. I went back for the check-up, and the doctors asked me, "Hey, what did you do? It's not there. What did you do? Did you see someone special?" I said," Yes, there was this old black lady. She pushed me to drink. And I had it." He said, "Bring that to me." He wanted to know the secret. No, you can't. You have to get it from that old lady. It belongs to her. I hadn't got it. She had got it. So, I asked to the lady, and she said, "It belongs to the land. Leave it where it is." That's the way life is. If you want anything, you go and ask for it. " *[Remote male participant]

For this participant, the spirituality associated with healing comes from and belongs to the land. To relinquish the bush medicine to the doctor would be subjecting it to a western medicalised inquiry that conflicts with that spirituality and with the holistic health worldview. The patient wanted to maintain the sacredness of his relationship with the country and its spirits. It could also be about protecting Aboriginal knowledge from appropriation by the western system which in the past has been highly exploitive. The old lady's response was a recognition of cultural protocols and affirmation of ownership in that, she did not have the authority to pass on the information. This highlights the tension between what is allowed to be public knowledge by Aboriginal people and what remains private.

Healing and the holistic health worldview were stressed several times, particularly by two interviewees who worked in health care. One of them emphasised that '*healing isn't just a physical thing' *rather it is very much related to patients' *'mental, emotional and spiritual' *state. This worker firmly believed that sometimes miracles do happen in life, and people could recover, even from serious illnesses like cancer. As one female participant who worked in an urban Aboriginal medical organization stated: "*the spirit world is an integral part of day-to-day life; yes, absolutely"*. The allusion to 'miracles' by the first respondent also supports the idea that bush medicine is spiritually-based. These participants reinforced the need to cater to the spirituality of Aboriginal patients as part of the healing process.

Many participants generally argued for accepting and communicating about the use of bush medicine with Aboriginal patients in their cancer treatment plan concurrently with western medicine. One participant who was a medicine-man expressed his feeling about someone benefitting from his medicine: *"if it worked... if either one (white-men medicine or the black-men medicine) that is good because it gives you a chance"*. To help in this way confirmed the man's healing ability, establishing his identity and status in the Aboriginal community as a healer and validating Aboriginal knowledge as having a legitimate place alongside western medical approaches. He also stated that he did not take money for bush medicine as he believed his ancestors would not approve, demonstrating his deep spiritual respect for his ancestral relations, a recurring theme in Aboriginal communities. Participants commonly noted that believing in the effectiveness of bush medicine is important: described by one participant as *"pure positive thinking"*. Another participant clarified that bush medicine and western medicine were not incompatible:

*"a lot of people say, 'Oh, yeah, that's just a lot of rubbish' and especially you will find doctors that say so...No, I'd never say, 'Discard conventional medicine and just concentrate solely on this', because I think it's got to complement each other, and if you've got those beliefs already... that this is gonna help you, it will (emphasizing). It may not cure you. It may not save your life, but it will help you, even if it's only in a mental or an emotional way of help. So, I really do believe that it would help, and have just having somebody there to go and smoke the house... to get rid of all the bad feelings. I mean that's ... a lot of these are very spiritual stuff that Aboriginal people have known for millennia," *[Urban female participant]

Bush medicines and traditional healing approaches are compatible with other complementary, alternative and integrative medicines, of which the use is increasing among patients with cancer, with the average prevalence rate of 31.4 percent in the Australian population[38]. This underscores the need for complementary therapies such as bush medicines and spiritual healing to be discussed with all patients undergoing cancer treatment. This was put into the context of ancient cultures by one participant:

*"Chinese have been practicing all this acupuncture, acupressure and all those sorts of things for thousands of years, and now it's all in vogue, so it's all right. It's the same thing with the bush medicines. Even meditation! and all these things. They are all of a sudden miraculously, 'Yes, they do work.' Well a lot of Aboriginal people, and old cultures have known that for so... long." *[Urban female participant]

As the Indigenous concept of wellness and hence healing is linked to their culture and spirituality, there is a need for health care providers to acknowledge and respect this component of Indigenous beliefs when providing health care.

Adverse reaction from biomedicine: *"Radiation and chemo nearly killed me"*

"*I know a couple of people who chose the bush medicine once they read up about chemotherapy and the two per cent of people that chemo cured, they took their chances with the bush medicine, and they are still going. It's either the quality of life or being sick from the chemo, that's what they weighed up." *[Rural female participant]

There are some Aboriginal people who use traditional medicine as an alternative to Western medicine. Both cancer patients and the family members felt some people get scared about the intensive procedures of common cancer treatments and their side-effects, influencing them to choose other options instead. As well, some patients did not cope with the side-effects of chemotherapy and radiation treatment and disliked having to spend long periods away from their family and home town. This was made more salient for Aboriginal women if they had the responsibility of taking care of their children and grandchildren, impacting on the choices made between using traditional healing and medicine so that they did not have to go away. One respondent summarized:

"*It's hard for a lot of people. So, they prefer to either go for bush medicine or not take the treatment, because they know that they are going to be away for a while from their family."*

The perception of some of the participants towards bush medicine was how well people were when they were taking it: *"She looked better when she took the bush medicine"*. These perceptions confirmed and validated the healing qualities of bush medicine as an alternative or as a complementary approach to Western medicine.

Last resort and desperation to try everything: *"at the end we were just clutching to hope"*

One urban female participant shared the story of her young relative who had died of cancer. The patient kept faith with the Western doctors, hoping that they were going to fix the cancer and seeing them as *'miracle-makers'*. However, when everything failed the family turned back to their traditional treatment which by then was too late as the cancer had advanced too far. After sharing her story, the participant admitted that "*really, they *(doctors) *are not miracle-makers and, we've got to start doing some stuff, too." *Attempting a range of different healing options to treat cancer, especially when Western medical treatments have not worked are not uncommon in many societies[39,40] and is another reason why some people turn to alternative medicine.

Having cancer caused fear and was often associated with fatalism about the likely consequences. Upon diagnosis, many people started thinking immediately about death and consequently panicked. This fear prompts them to desperately try everything to cure the disease. One participant said:

"*I would try different treatments. I would try what I have heard works. I would definitely try the hospital, what they had to offer. I would try... if I had heard of a good bush medicine that could fix it, I would try that. Ye.., I wouldn't be hesitant in using alternative medicines at all, whether it be from the Aboriginal bush medicine or from somewhere else." *[Urban female participant]

Although this participant reported not knowing much about bush medicine because she grew up in urban areas, she said she would give anything a go if somebody said that it could work.

### Reasons why bush medicine was not used

Many respondents did not use bush medicine or did not talk about the use of bush medicine during the interviews. For many, it was not because they did not want to use bush medicine, but rather that they did not have access to the source, got confused about what would be better for them to use, or were unsure about the process of taking it.

#### Not easy to get

Many participants, especially Aboriginal people who lived in the city and in the rural towns admitted that it was hard to get bush medicine, as most traditional healers lived in rural and remote areas. This meant that they either had to travel away from where they lived or organise for the traditional healer and supplier of bush medicine to travel to where they lived; both a time-consuming and expensive exercise. These issues restricted their choice of using bush medicine. Some people also explained that although they wanted to use bush medicine, they did not know who, how and where to contact a traditional healer.

One participant when asked if they had taken any bush medicine replied:

*"No, no. No, I haven't had any. No, I got to go up to Wiluna and get some." *[Rural female participant]

It should be noted here that a healer has to be authorized to be able to practice and prescribe bush medicine. As one participant described:

*"The 'ok' to use it. You just can't go and use it. He told me that I could go ahead and get the medicine, and prepare them, and use it. Otherwise, in our ways, you can't just use it unless anybody given the 'ok' to you to use it. So, he gave me the 'ok' to use it." *[Urban female participant]

Being given the authority to collect the plant used for treating cancer also involved being trusted enough to be told where to harvest it, how to prepare the medicine and how and when to take it. For this to happen requires that the person has a good relationship with the healer, who would not hand over his/her knowledge over lightly.

Urbanized Aboriginal people: *"... we are urban Aboriginal, we are not traditional"*

A devastating effect of colonisation was the alienation and disconnection of Aboriginal people from their land, their cultural heritage and traditions. Being taken away from their family and raised elsewhere on missions or placed in non-Aboriginal families was traumatic for those who were removed. The separation from their traditional country and families and the relocation to urban and regional centres meant that for some there was a loss of cultural knowledge, language and tradition. Some respondents admitted that they had lost their connection with their traditions and culture, while others said that they continued to visit their homeland occasionally, for funerals or other ceremonies. Participants who grew up in Western society and had been exposed to Western education had access to modern technologies and information systems and a reasonable knowledge of the cancer that troubled their family member. Many of these people did not try to look for bush medicine and traditional healing. As one of the participants said:

*"We were born into ... a society that were fully functional at that time... we are urban Aboriginal, we are not traditional. We have access to information, technology, whatever. We didn't have any Aboriginal remedies... or anything like that..." *[Urban female participant]

However, not all urban Aboriginal people subscribed to this view. Traditional beliefs and practices persist amongst many urban Aboriginal people and may become visible only when it affects those who are close[41]. 18 participants reported that they had connections with some traditional practices, but not in a very strong way. For example, use of bush medicines was one thing some of the families were practicing despite limited knowledge and access to other traditional healing practices.

Another reason given by some participants for foregoing traditional practices was religious beliefs. Christianity was imposed as part of the colonizing process and with this came restrictions upon Aboriginal peoples' life-style and values system[41]. Many Aboriginal people were exposed to Christianity within the missions, places where they were forced to leave their Aboriginal beliefs, culture and traditional rituals behind. Those directly affected by missions and subsequent generations, who have grown up in a "Christian" environment, may regard traditional Aboriginal beliefs as akin to paganism and thus discourage their use. In the words of one participant:

*"We didn't use traditional medicine or anything like that. Because we are not traditional Aboriginal, and our family was Christian based, and so...We put our trust on God." *[Urban female participant]

Dilemma of usage: *"I was a bit worried taking any of that..."*

Secrecy and mystery abound in the Aboriginal community about the use and availability of bush medicine. This inevitably means poor availability of accurate information regarding its actual use. As Western medicines usually have detailed prescriptive and side effect information available, this created an expectation among some Aboriginal people for similar processes and information being available for bush medicine. As one participant said:

*"I was a bit worried taking any of that because none of them could tell me exactly how much, what quantity to take and I was worried about that...." *[Rural female participant]

Another participant from the rural area said that she tried bush medicine but had severe reactions (rash and urine infections) so she stopped it. She wanted to just stay on bush medicine provided more accurate information and guidance was given to her. The conflict between the use of western and traditional healing meant patients had to make choices, presumably based upon their relative confidence in what each treatment would offer: *"I tried *[bush medicine], *but, yeah, I think it reacts with all my tablets I'm taking."*

## Discussion and Conclusions

There has been little study of the role of bush medicine and other traditional healing in contemporary Aboriginal society in Australia, and very little about the use of traditional medicine in cancer treatment: what information is available is anecdotal. The desire to use traditional medicines among Aboriginal patients is still widespread, even for a serious disease like cancer. Aboriginal participants in the study acknowledged traditional healing practices and use of bush medicines as important aspects of cancer treatment. Bush medicine has spiritual significance for Aboriginal people as it is natural, comes from the land, connects to identity and spirituality and plays an important role in people's health and wellbeing. Bush medicine is also connected to the holistic world view in such a way that the interplay between the physical, emotional, social and spiritual aspects is crucial in attaining wellbeing. Whereas hospitals and Western medical systems are representative of the dominant society reminding Aboriginal people of their loss of cultural knowledge, access to the traditional healing system, bush medicine and other healing processes repairs some of the damage inflicted by colonisation. The opportunity to access traditional knowledge through other groups who have retained this knowledge can be reassuring for Aboriginal people with cancer.

People often turn to spirituality in dire situations, and this is the same for cancer which is often regarded as a death sentence[24]. Aboriginal people with cancer who were at the end-of-their life phase and were away from their home indicated that they wanted to go home. On returning to their home they then incorporated bush medicine and other traditional healing processes into their treatment. Findings from the study revealed that the use of alternative medicines and approaches in cancer treatment often brought comfort and peace to the patient. Unlike Western medical treatments, traditional healing is embedded in holism and challenges the biomedical system that Aboriginal patients have to deal with. The use of traditional medicines and healing can empower patients in the health process, creating possibilities for positive outcomes.

This study revealed that in Aboriginal communities the cause of cancer is attributed to a number of different things; stress and/or the influence and impact of the dominant culture and sometimes cultural causes. It is often interpreted as a "white-man's disease" and linked to colonisation. These factors can create a hesitation to use bush medicine because of the pressure and expectation to engage with the dominant health system and the well-established biomedical model. On the other hand, bush medicine is considered culturally safe, a practice connected to Aboriginal ways of being and doing. Applying bush medicine and engaging with an Aboriginal healer provides comfort from a cultural perspective that is healthy and healing for the spirit. This explains why some Aboriginal Australians see bush medicine as a non-stressful alternative treatment for a disease that may be attributed to stress.

For health care providers, it is important that they have an appreciation and understanding of Indigenous belief systems in relation to health care, and work to incorporate this understanding into their service delivery. One way to do this would be to adopt a family-centred, integrative approach that works with the individual in concert with their familial and cultural support. Applying this type of approach not only respects Aboriginal people's choice to utilize bush medicine as a part or whole of cancer treatment and their overall search for health and wellbeing, but recognizes and begins the journey of working with Indigenous epistemology and ways of doing.

It is also of value for practitioners to know that their patients are taking bush medicine because there can be potential risks involved in using both. Plants, leaves and trees used in making bush medicines may be bioactive and can have physiological, emotional and psychological effects. A well-known example of a herbal drug that interacts with biomedicines is St. John's Wort, Hypericum perforatum, which is a traditional European herbal drug used to treat mild depression that interacts with a wide number of biomedicines, including anti-retroviral drugs, oral contraceptives and warfarin[42].

Another risk that medicinal plants may pose is that they often may not be safe to use. Effects may not be immediate, and the potential toxicity of plants may be hidden to traditional healers. Many of the plants used traditionally by Aboriginal people in Australia have not been studied phytochemically, thus this is an unknown area. Thus, acceptability and understanding of the use of these medicines would provide a rationale for dealing with such issues. Further exploration of these issues may be needed but this needs to start with clinicians being alert to the possibility of use of bioactive agents that are not prescribed.

Recognition and understanding of the use of traditional medicine and healing system can boost the confidence of Aboriginal people to access mainstream services and it will definitely improve the delivery of health services to Aboriginal communities. Both medicine needs to be supported and developed with enduring research so that the therapeutic value of traditional medicine can be judged and understood. The growing popularity and use of complementary and alternative medicine world-wide may assist and support the improvement and sustainability of Aboriginal Traditional medicine and healing in Australia.

## Competing interests

The authors declare that they have no competing interests.

## Authors' contributions

SS participated in the project's design, carried out the data collection and analysis for this project, prepared the initial draft. RB contributed to preparing the initial draft and commented upon drafts of the manuscript. DB was involved in writing and commented upon drafts of the manuscript. SCT coordinated the whole project, participated in the design and assisted with the conduct of the study and writing. All authors read and approved the final manuscript.
